# A Real-World Grounded Usability Evaluation of VR Interaction Techniques Across Discrete, Spatial, and Continuous Tasks

**DOI:** 10.3390/s26216913

**Published:** 2026-10-31

**Authors:** Jeong Hyeon Lee, Minwoo Kim, Hyeon Seo, Kangsoo Kim, Sukwon Lee, Changgu Kang

**Affiliations:** 1 Department of Computer Science and Engineering, Gyeongsang National University, Jinju 52828, Republic of Korea; ljhyun0821@gnu.ac.kr (J.H.L.); rlaalsdn4560@gmail.com (M.K.); hseo0612@gnu.ac.kr (H.S.); 2 Department of Electrical and Software Engineering, University of Calgary, Calgary, AB T2N 1N4, Canada; kangsoo.kim@ucalgary.ca; 3 Department of AI Content Engineering, Kangwon National University, Wonju 26403, Republic of Korea

**Keywords:** virtual reality interaction, usability evaluation, task-based interaction, real-world baseline

## Abstract

With the growing use of virtual reality (VR), interaction techniques need to be evaluated across different task demands and in relation to real-world performance. This study compares four VR interaction techniques—Direct Controller, Hand Tracking, Raycasting, and Wrist Rotation—across discrete (text typing), spatial (tangram manipulation), and continuous (slider control) tasks. Twenty participants completed corresponding tasks in both real-world and VR environments. Task completion time and three questionnaires—the Reality-Comparison Questionnaire (RCQ), Technique-Appropriateness Assessment Questionnaire (TAAQ), and User Experience Questionnaire (UEQ)—were used to assess performance and user experience. Participant-level repeated-measures ANOVAs and Holm-adjusted paired tests were conducted. The results showed task-dependent differences among interaction techniques. Direct Controller was faster than Hand Tracking and Raycasting for tangram manipulation, while their typing-time differences were not significant after correction. Direct Controller also received higher agency ratings than Raycasting in typing and tangram. Raycasting had the lowest mean slider completion time, although its difference from Wrist Rotation was not significant after correction, while Wrist Rotation was also associated with higher physical fatigue. Hand Tracking received higher immersion and embodiment ratings than Raycasting in typing and tangram but showed longer completion times in the continuous task. Overall, the findings indicate that interaction performance and user experience vary with task characteristics, highlighting the importance of task-aware selection of VR interaction techniques.

## 1. Introduction

As virtual reality (VR) technology becomes increasingly widespread, a variety of interaction techniques have been explored to enhance user convenience and accuracy in immersive environments. Among these, controller-based interaction remains the most common, offering physical buttons, haptic feedback, and precise manipulation capabilities [1]. Raycasting is another widely adopted technique, enabling users to point and select virtual objects using a simulated light beam [2]. Hand tracking, which captures the user’s hand gestures without requiring physical controllers, has gained significant attention as a promising alternative [3]. Additionally, gaze-based targeting has been shown to reduce cognitive load during object selection tasks [4].

Other emerging interaction techniques include wrist rotation-based input for 3D object selection [5], as well as hybrid approaches that combine head, eye, and controller tracking for rapid and precise selection in dense 3D scenes [6]. Researchers have also investigated the use of smartphones and smartwatches as input devices in augmented reality [7] and the combination of gaze tracking with virtual surfaces for complex environment interaction [8]. Hands-free methods such as voice commands [9] and electromyographic (EMG) input based on forearm muscle activity [10] have also been actively studied.

Despite the diversity of input methods, most existing studies compare interaction techniques within a single task type. While this is effective for identifying the most efficient method for a specific activity, it limits our understanding of how user experience changes across different task categories. Moreover, evaluations are often conducted solely in VR, without grounding them in real-world performance. As a result, current research offers limited insight into how task type and input modality jointly influence user experience when VR interactions are compared against real-world baselines. Without such grounding, it becomes difficult to interpret whether observed performance differences reflect meaningful interaction advantages or artifacts specific to virtual environments.

To address these gaps, we designed an experiment comparing participants’ real-world performance with corresponding VR tasks using three widely adopted baseline interaction techniques—Direct Controller, Hand Tracking, and Raycasting—along with a task-specific technique, Wrist Rotation, introduced to examine one-dimensional continuous control.

Direct Controller and Hand Tracking are widely adopted interaction methods natively supported by commercial HMDs. Raycasting, as an indirect interaction technique, is also commonly employed for UI navigation and remote object manipulation in VR. In our previous systematic mapping study of XR interaction research [11], Direct Controller, Hand Tracking, and Raycasting were identified as the three most frequently investigated interaction techniques across representative VR task categories. Building upon these findings, the present study experimentally evaluates these techniques together with Wrist Rotation in a controlled user study. Wrist Rotation, while less frequently adopted as a general-purpose interaction technique, has been explored as an efficient input mapping for one-dimensional continuous control, such as UI navigation and parameter adjustment [5,12]. In this study, it was intentionally included to examine task–technique alignment in continuous control tasks, rather than to serve as a baseline competing with the other techniques across all task types.

Following the task taxonomy established in our previous systematic mapping study [11], the interaction tasks were categorized into three representative VR task types: discrete (text typing), spatial (tangram puzzle manipulation), and continuous (slider-based video control). The spatial task was designed to involve object selection, translation, rotation, and placement, reflecting common 3D manipulation demands in VR. Each category represents distinct input characteristics, including symbolic entry, spatial manipulation, and continuous adjustment.

While our previous work established a literature-based taxonomy of VR task types and identified widely adopted interaction techniques, the present study extends that work by experimentally comparing these techniques across the three representative VR task categories using corresponding real-world tasks as performance baselines.

This study makes the following key contributions:

- We evaluate VR interaction methods by comparing their performance against corresponding real-world tasks, providing a participant-specific baseline for interpretation.
- We systematically assess widely adopted baseline techniques—Direct Controller, Hand Tracking, and Raycasting—together with a task-specific technique, Wrist Rotation, across tasks representing discrete, spatial, and continuous interaction types.
- We identify technique–task suitability patterns and offer design implications for selecting interaction methods tailored to specific VR application contexts.

## 2. Background and Rationale for Technique and Task Selection

Numerous studies have compared interaction techniques in virtual reality (VR), augmented reality (AR), and mixed reality (MR) to improve user performance and interaction efficiency. Previous research has evaluated a wide variety of input modalities, including controller-based interaction, hand tracking, raycasting, eye tracking, voice input, smartphones, and wearable devices, across diverse application domains.

Our previous systematic mapping study [11] reviewed 45 VR/AR/MR interaction studies published over the past decade and analyzed the relationships among task types, interaction techniques, and evaluation measures. Building upon these findings, the present study focuses on experimentally validating representative interaction techniques using controlled user experiments with real-world baselines.

### 2.1. Selection of Interaction Techniques

Based on the findings of our previous mapping study [11], Direct Controller, Hand Tracking, and Raycasting were identified as the three most frequently adopted interaction techniques across representative VR tasks. Accordingly, these techniques were selected as the baseline interaction methods in the present study. In addition, Wrist Rotation was included as a task-specific interaction technique for one-dimensional continuous control.

**Direct Controller.** Direct Controller is the most widely adopted interaction technique in commercial VR systems because it provides reliable tracking, physical buttons, and haptic feedback for precise object manipulation. Sowell et al. [1] reported more stable performance and higher user satisfaction than hand tracking in industrial training. Wang et al. [13] demonstrated faster and more accurate object manipulation than indirect interaction methods. Wentzel et al. [2] also reported superior menu interaction performance.

**Hand Tracking.** Hand Tracking enables natural interaction by directly reflecting users’ hand movements without requiring handheld controllers. Previous studies have consistently shown that hand tracking increases immersion and the sense of embodiment, although precise manipulation remains challenging. Voigt-Antons et al. [3] observed improved immersion but reduced interaction precision. Maurer et al. [14] similarly reported stronger ownership but longer task completion times.

**Raycasting.** Raycasting is an indirect interaction technique that enables efficient remote object selection and menu interaction. Luro and Sundstedt [4] compared eye-tracking- and hand-controller-based aiming. Wentzel et al. [2] found raycasting preferable for large menu interaction. Ablett et al. [15] demonstrated effective interaction with occluded virtual objects.

**Wrist Rotation.** Unlike the previous three baseline techniques, Wrist Rotation was included specifically for continuous control tasks. It has been applied to one-dimensional parameter adjustment and menu navigation. Di Domenico et al. [12] proposed a radial menu controlled by wrist rotation, while Wu et al. [5] integrated wrist rotation with raycasting for dense virtual environments.

Overall, these findings motivated the selection of Direct Controller, Hand Tracking, and Raycasting as representative baseline techniques and Wrist Rotation as a task-specific interaction method in the present study.

### 2.2. Selection of VR Task Categories

Our previous systematic mapping study [11] categorized VR interaction tasks into three representative groups according to their interaction characteristics: discrete, spatial, and continuous. This taxonomy provides a practical framework for selecting representative experimental tasks while maintaining broad coverage of common VR interaction scenarios.

**Discrete Tasks.** Discrete tasks require explicit selection or symbolic input among distinguishable alternatives. Representative examples include target selection, menu selection, and virtual keyboard text entry.

**Spatial Tasks.** Spatial tasks involve object manipulation in three-dimensional space, including translation, rotation, alignment, and placement. Representative examples include tangram puzzle assembly, object arrangement, and remote manipulation tasks.

**Continuous Tasks.** Continuous tasks require sustained control through continuous user input and real-time feedback. Examples include object tracking, path following, drawing, and continuous parameter adjustment.

Based on this taxonomy, the present study selected text typing, tangram puzzle manipulation, and slider-based video control as representative experimental tasks for the discrete, spatial, and continuous categories, respectively. These tasks enable systematic comparisons of interaction techniques under substantially different interaction demands while maintaining consistency across experimental conditions.

## 3. Methodology

### 3.1. Apparatus and Interaction Techniques

The experiment was conducted using a Meta Quest 3 standalone head-mounted display (HMD) [16], operating in untethered mode at a refresh rate of 90 Hz. Input was provided through the Meta Quest Touch Plus controllers [17] and the headset’s built-in hand tracking system, which utilizes internal cameras. The virtual environment was implemented using the Unity engine (version 2023.2.20) [18], and all interactions were developed using the Meta XR All-in-One SDK (v72.0.0) [19]. The final application was deployed as a standalone build, allowing the experiment to be run directly on the HMD without requiring an external PC connection.

We implemented three widely adopted baseline interaction techniques—direct controller, hand tracking, and raycasting—together with an additional task-specific technique, wrist rotation. The baseline techniques were implemented using the default prefabs (preconfigured, reusable Unity GameObject templates bundling components and their settings) provided by the Meta XR All-in-One SDK, while wrist rotation was custom-developed by extending the SDK’s input handling functions. Direct controller, raycasting, and wrist rotation operate via the Meta Quest Touch Plus controllers, whereas hand tracking relies on the headset’s built-in cameras.

Direct controller renders a virtual controller model and enables object grasping through the grip button, as well as UI interaction via direct contact using the controller tip or fingertip. Hand tracking represents users’ hands with a tracked hand mesh, supporting direct manipulation and pinch-based object selection. Raycasting projects a pointer ray from the controller to support remote selection and manipulation using trigger input. Wrist rotation uses roll and pitch for the split typing keyboard and a continuous angular mapping for slider control; the implemented mapping provides no positional input for general 3D spatial manipulation.

### 3.2. Task Design

Interaction tasks were classified into three categories—discrete, spatial, and continuous—based on input characteristics commonly encountered in immersive VR applications. Each task was implemented in both real-world and VR environments, enabling within-participant comparisons using real-world performance as a baseline. This categorization follows prior VR research, where text typing evaluates accuracy- and speed-sensitive discrete interactions [20,21], tangram puzzles represent spatial manipulation involving object placement and rotation [22,23,24], and slider-based controls (e.g., media playback adjustment) examine continuous input [25,26,27].

**Task Selection Rationale.** The specific tasks were selected to serve as representative and well-established exemplars of discrete, spatial, and continuous interaction demands in VR. Rather than introducing novel or overly abstract tasks, we chose tasks that have been widely used in prior VR and AR research, allowing results to be interpreted in relation to existing literature and real-world interaction behaviors. Text typing was selected as a canonical discrete task, as it requires explicit symbolic input with clear accuracy and speed constraints [20,21,28,29]. Tangram puzzle assembly was chosen to represent spatial interaction because it naturally integrates object selection, translation, rotation, and placement—core components of 3D manipulation commonly encountered in VR applications [22,23,24]. Slider-based control was adopted as a representative continuous task, as it involves sustained one-dimensional adjustment with continuous feedback, directly reflecting common UI operations such as media control and parameter tuning [25,26,27].

**Figure 1:**
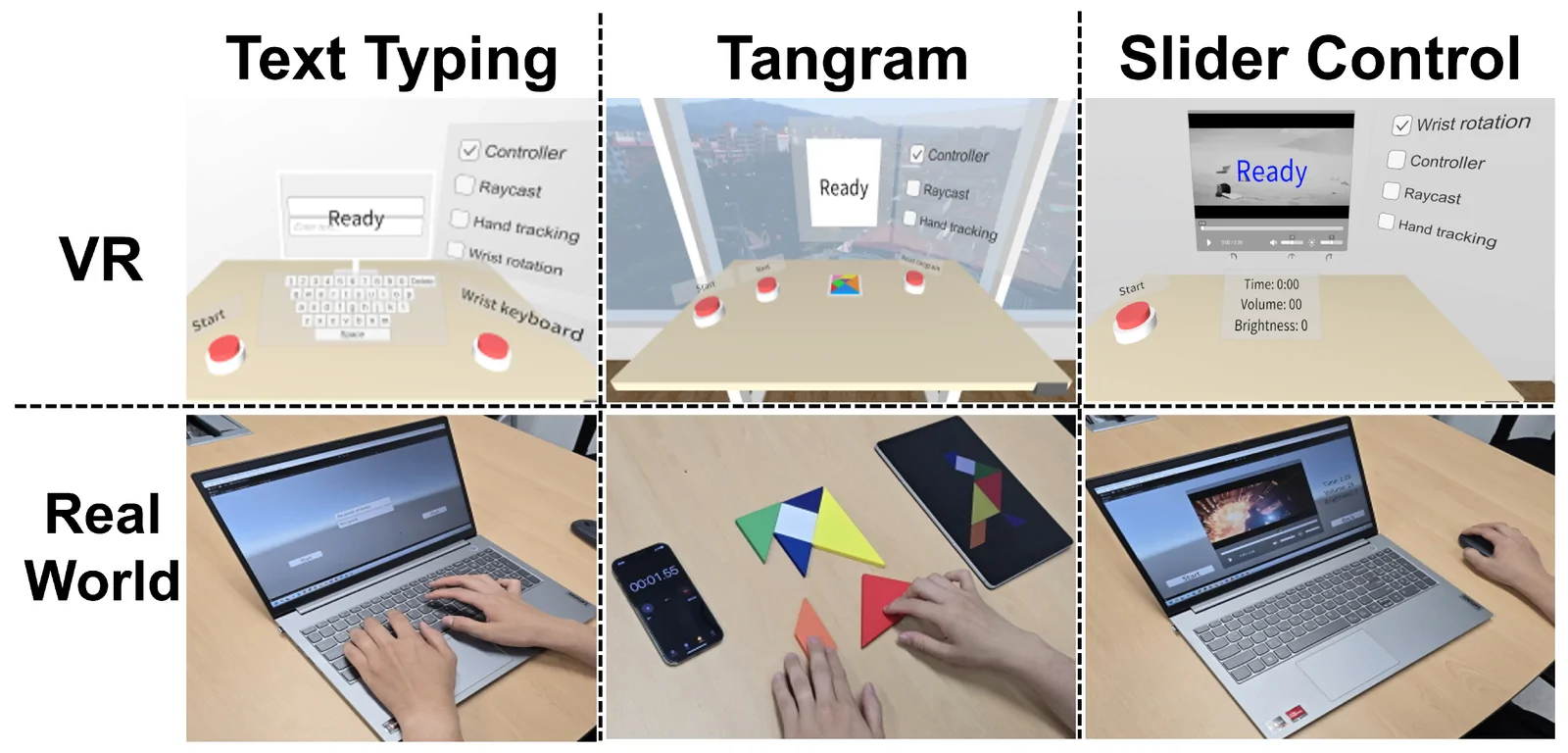
Experimental setups for the three tasks in VR (**top row**) and real-world environments (**bottom row**), arranged from left to right as: text typing, tangram, and slider control.

Figure 1 shows the experimental setups for the three selected tasks—text typing (discrete), tangram (spatial), and slider control (continuous)—in both VR (top row) and real-world (bottom row) environments.

**Rationale for Precision-Demanding Spatial Task.** Although spatial interaction tasks in VR are often associated with free-form object manipulation, many real-world and application-driven VR scenarios require a high level of precision in object placement and alignment, such as assembly training, object docking, and industrial or medical simulations, where inaccurate positioning or orientation directly affects task success [30,31]. To reflect these practical demands, we intentionally selected a spatial task that requires accurate selection, translation, rotation, and placement.

The tangram task was therefore employed not as a cognitive puzzle but as a controlled precision-demanding spatial manipulation task, as has been done in prior VR studies focusing on object placement and spatial coordination [22,23]. By restricting the task to easy-level puzzles, the task emphasizes interaction accuracy and motor control rather than problem-solving ability. This design allows the strengths and limitations of different interaction techniques to be revealed under accuracy-sensitive spatial conditions, which are common in real-world VR applications.

#### 3.2.1. Discrete Task Configuration (Text Typing)

Short sentence typing was implemented using phrases from MacKenzie and Soukoreff’s set (lowercase only, 19–21 characters) [29], split into separate sets for real-world and VR conditions. Participants typed one sentence per trial, giving three trials (one per length) per technique. Text and completion time were recorded. All techniques used a QWERTY keyboard; wrist rotation used a split keyboard navigated via roll and pitch angles (gain 1.3).

#### 3.2.2. Spatial Task Configuration (Tangram)

A tangram task used 20 “easy” puzzles from the Animals and Miscellaneous categories [32]. Five were used in real-world and 15 in VR conditions, with colors matched to physical pieces. Direct controller, hand tracking, and raycasting were used; wrist rotation was excluded because the implemented mapping provided no positional input. To minimize cognitive load unrelated to interaction performance, only easy-level puzzles were used; equivalence of difficulty between the different real-world and VR puzzle sets was not established.

#### 3.2.3. Continuous Task Configuration (Slider Control)

A video player UI with sliders for playback position, volume, and brightness was implemented. Each parameter had 25 randomized target values, with five instructions in the real world and 20 in VR. Direct controller, hand tracking, and raycasting interacted with sliders directly or via rays. Wrist rotation adapted the continuous mapping method to rotate a virtual knob controlling each slider.

### 3.3. Experimental Procedure

A total of 20 participants took part in the experiment. All participants were between the ages of 20 and 25 (M=22.65, SD=1.71), with 14 identifying as male (70%) and 6 as female (30%). Regarding prior experience with VR devices, 7 participants (35%) reported no prior experience, 11 (55%) reported limited experience, and 2 (10%) reported extensive experience. Participants who normally wore glasses completed the experiment while wearing them to minimize visual discomfort.

The study was approved by the Institutional Review Board of Gyeongsang National University (IRB No. GIRB-A25-NY-0048). Written informed consent was obtained from all participants prior to participation.

**Figure 2:**
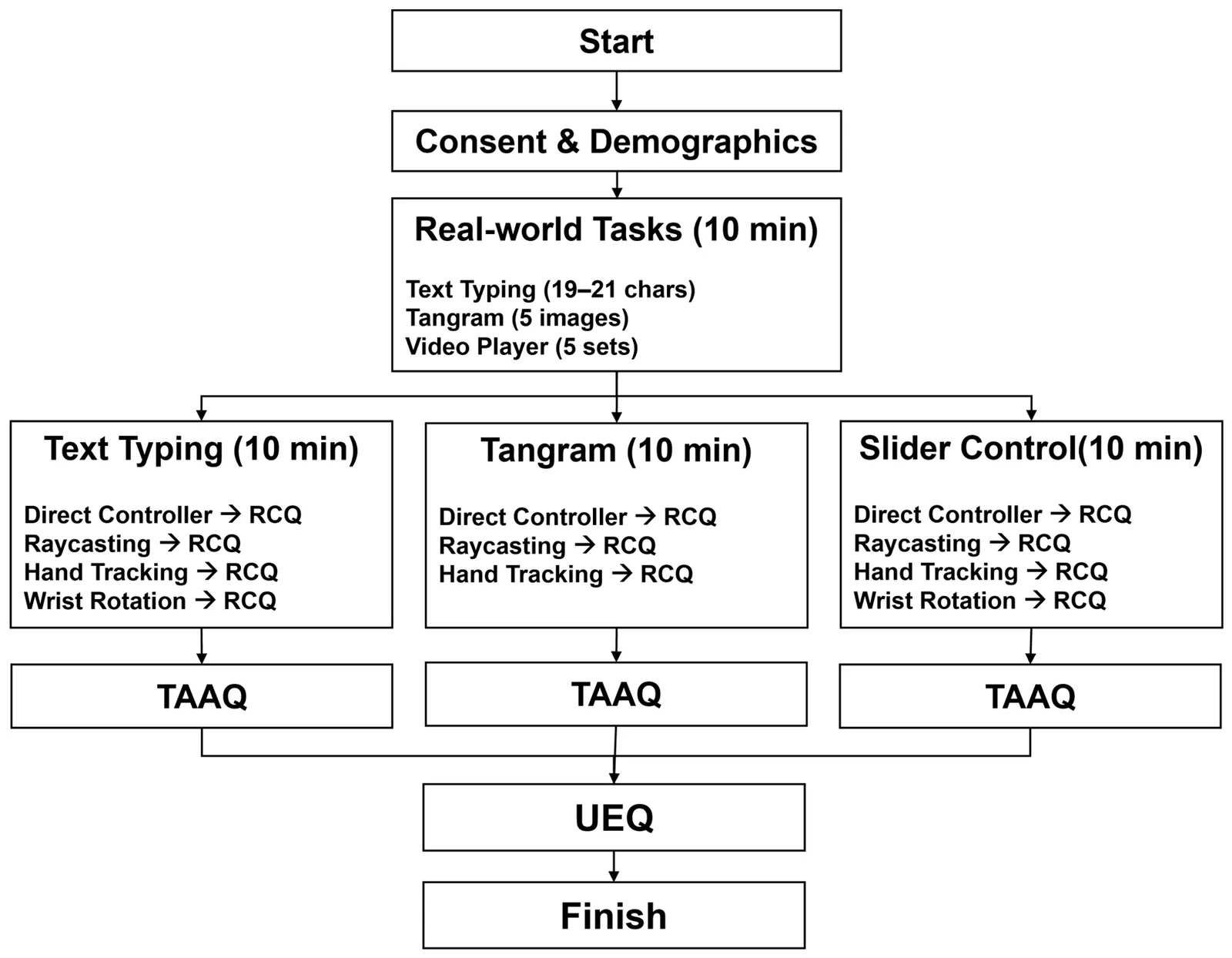
Overview of the experimental procedure. The real-world-first path is illustrated; the environment-phase order was reversed for half of the participants. Technique labels identify conditions rather than a fixed execution order.

Figure 2 illustrates the overall experimental flow. The procedure was verbally explained before the experiment, after which participants completed a demographic questionnaire.

Environment order was balanced across participants: 10 completed the real-world phase before VR, and 10 completed VR before the real-world phase. Environment order was balanced across participants: 10 completed the real-world phase before VR, and 10 completed VR before the real-world phase. This environment order and the manual timing procedure for real-world tasks were retrospectively confirmed by the experimenters. The real-world tasks were performed in the following order: text typing, tangram, and slider control.

For the real-world text typing task, participants entered the presented phrases using a laptop keyboard. In the tangram task, participants manually arranged physical puzzle pieces to match a target image. For the slider control task, participants used a laptop and mouse to adjust playback position, volume, and brightness. For all real-world tasks, the experimenter observed participants and measured each trial from start to completion using a timer. VR completion times were recorded automatically in Unity.

Participants wore the HMD for the VR phase. Prior to each VR task, participants received verbal instructions and completed a brief practice session to familiarize themselves with the interaction technique. Participant-specific VR technique sequences could not be verified from the available reanalysis files. Each trial began after participants pressed a “Start” button, followed by a 3-s countdown.

**Figure 3:**
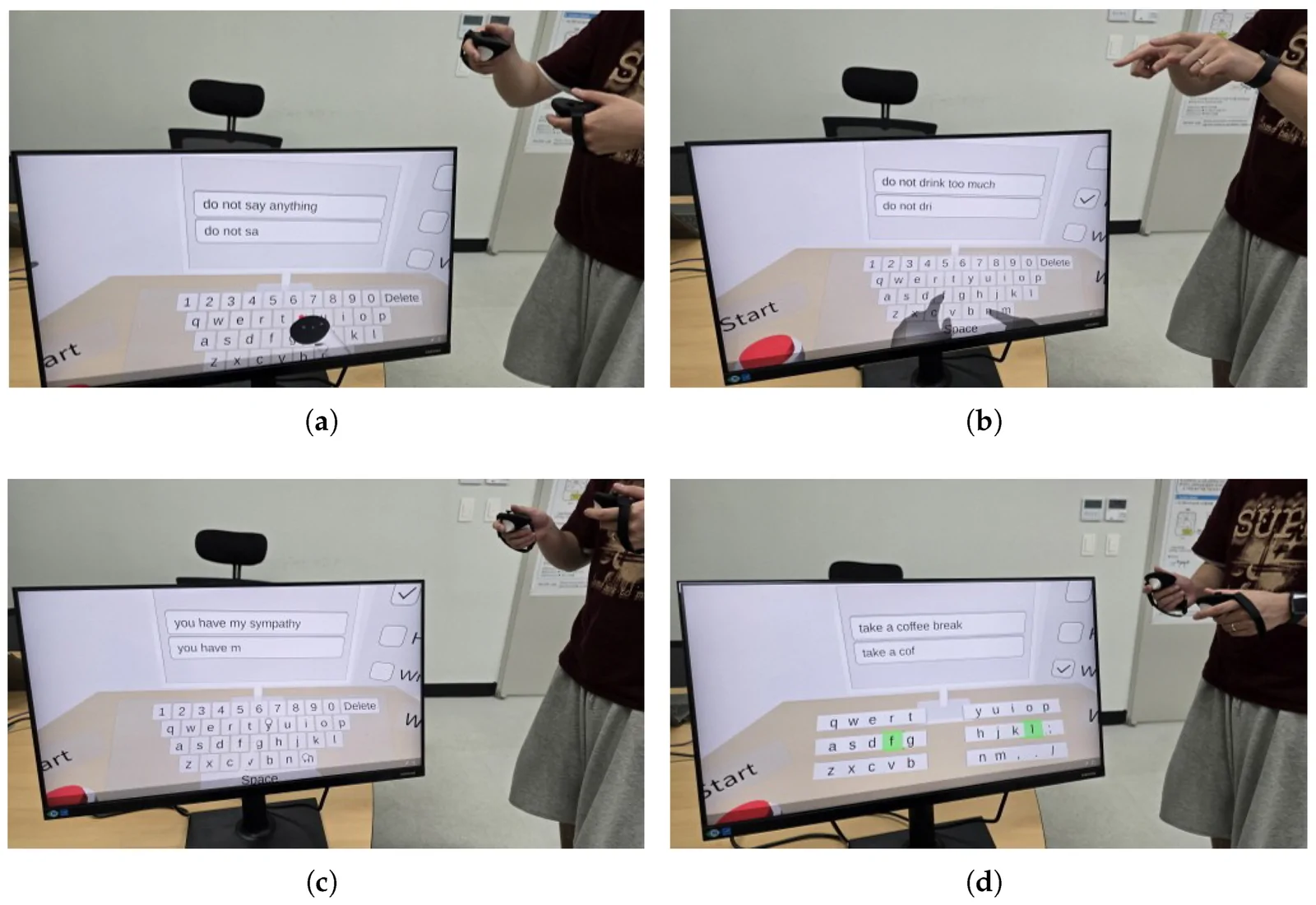
Text typing task in VR using four interaction techniques: (**a**) Direct Controller, (**b**) Hand Tracking, (**c**) Raycasting, and (**d**) Wrist Rotation.

**Text Typing.** A sentence was displayed in front of the participant. Once the sentence was typed correctly using the virtual keyboard, the input time was automatically recorded and the next sentence appeared. Three sentences were presented in ascending length order (19, 20, and 21 characters). All four interaction techniques—Direct Controller, Hand Tracking, Raycasting, and Wrist Rotation—were evaluated (Figure 3).

**Figure 4:**
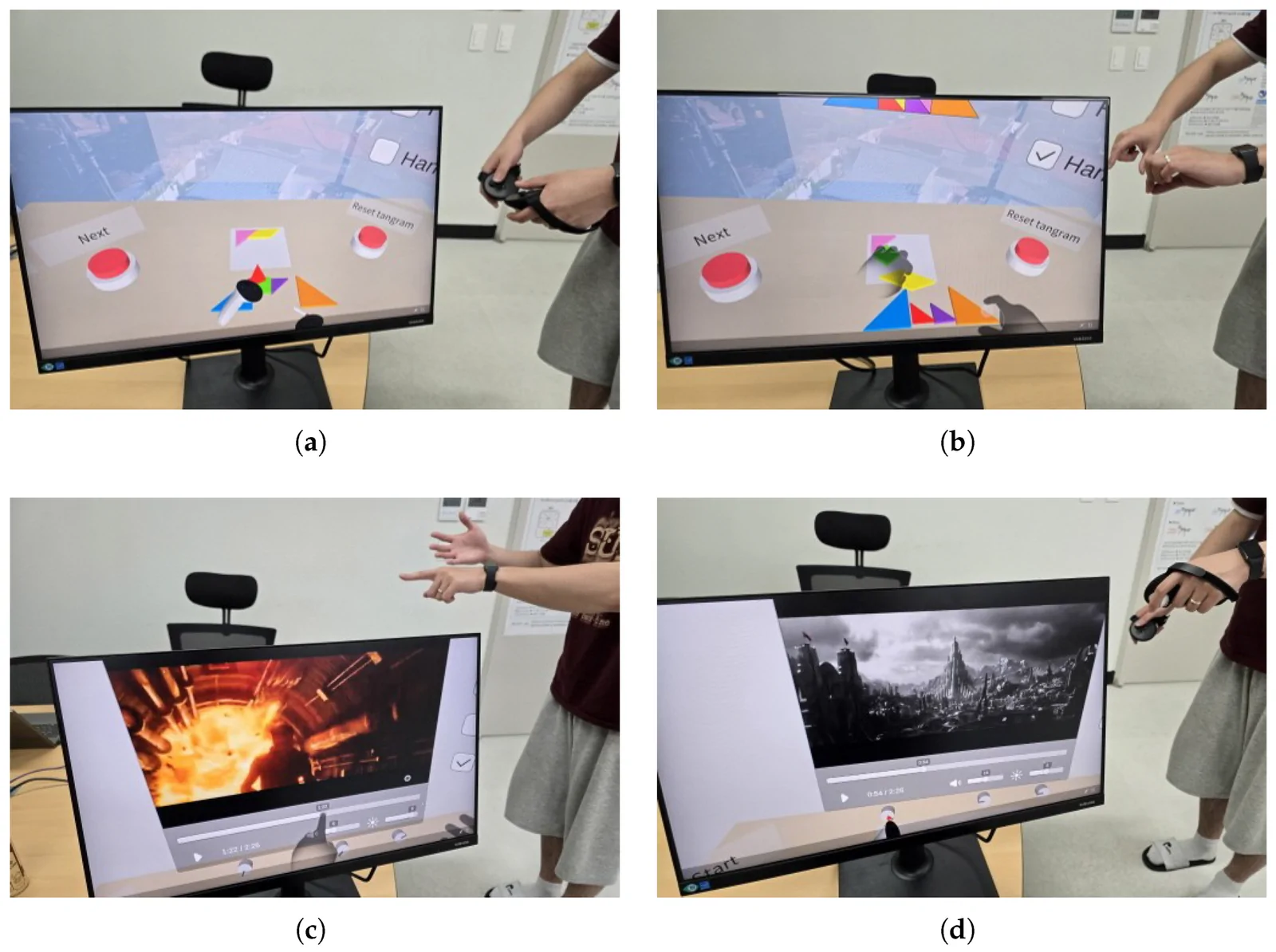
Example VR experimental setups: (**a**) Spatial task (tangram) using Direct Controller, (**b**) Spatial task (tangram) using Raycasting, (**c**) Continuous task (slider control) using Hand Tracking, and (**d**) Continuous task (slider control) using Wrist Rotation.

**Tangram.** A tangram target image was displayed in front of the participant. Participants manipulated virtual pieces until Unity accepted their positions within the implemented tolerance, then pressed a “Done” button to record task completion time. Five randomly selected images were presented. Wrist Rotation was excluded because its implemented mapping provided no positional input. The remaining three techniques—Direct Controller, Hand Tracking, and Raycasting—were evaluated (Figure 4).

**Slider Control.** Target values for playback position, volume, and brightness were displayed in the participant’s field of view. Participants adjusted the sliders until all three numerical values exactly matched the targets. Upon successful alignment, task time was recorded and the next instruction appeared. Five randomly generated target sets were presented. All four interaction techniques were evaluated (Figure 4).

After each task–technique condition, participants completed the RCQ ratings of perceived agency, directness and naturalness, and hand-related experience, as operationalized in Section 3.4.2. Upon completing all interaction techniques for a given task, participants indicated their preferred technique for that task. At the end of the experiment, participants completed the User Experience Questionnaire (UEQ) to assess overall user experience for each interaction technique. The entire experiment lasted approximately 40 min per participant, and VR completion times were automatically recorded, while real-world times were measured by the experimenter using a timer.

### 3.4. Usability Evaluation Methodology

#### 3.4.1. Task Performance Measurement Procedure

Task completion time was used as the primary objective performance metric and was compared within participants across the real-world baseline and VR conditions, with different timing procedures as described in Section 3.3. Completion time was selected because it provides a direct and comparable measure of interaction efficiency across heterogeneous tasks and input modalities. Completion time may include delays associated with errors, corrective actions, or interaction difficulty. It cannot identify their individual contributions or quantify error frequency; the objective comparisons therefore concern time to completion rather than task-specific accuracy. Wrist Rotation, whose implemented mapping provided no positional input, was excluded from the spatial task and applied only to the discrete and continuous tasks.

VR task duration was computed as the difference between system timestamps recorded at task initiation and task completion; real-world duration was measured manually by the experimenter. In VR, task start was triggered by a system-generated countdown following participant confirmation, and task completion was recorded automatically or via an explicit “Done” button, depending on the task.

**Text Typing.** One sentence was presented per trial, with lengths of 19, 20, and 21 characters in ascending order. For each sentence, the presented text and completion time were recorded, giving three trials per interaction technique.

**Tangram.** Five tangram images from the Animals and Miscellaneous categories were randomly selected and presented one at a time. Task time was measured from task initiation until the participant pressed the “Done” button indicating completion of the arrangement. This task was repeated five times per applicable interaction technique.

**Slider Control.** Five instruction sets specifying target values for playback position, volume, and brightness were randomly presented. Task time was recorded from task initiation until all three numerical slider values exactly matched their targets. This task was repeated five times per interaction technique.

Saved times and stimuli do not identify errors or corrections preceding successful completion. Error rates and placement deviations therefore cannot be reconstructed from these records.

#### 3.4.2. Questionnaire Instruments

User experience and perceived interaction quality were assessed using a three-stage questionnaire protocol designed to capture complementary subjective dimensions at different points of the experiment.

The RCQ was designed to capture how closely each VR interaction method approximated real-world interaction in terms of agency, immersion, and embodiment.

After each task–technique condition, participants completed the six-item, five-point Reality-Comparison Questionnaire (RCQ; Table 1). Its operational categories were perceived agency (intention and control), immersion (directness and naturalness), and embodiment (hand resemblance and perceived hand location). Longo et al. [33] and Wang et al. [13] provide conceptual grounding for these themes. The study-specific item pairs do not replicate the full questionnaires in those studies or directly quantify real-world equivalence.

Second, after completing all interaction methods for a given task, participants filled out the Technique-Appropriateness Assessment Questionnaire (TAAQ). Rather than rating each technique independently, the TAAQ employed a forced-choice format in which participants selected the single interaction method that best matched each item. This design emphasizes relative suitability between techniques and directly supports technique–task matching analysis. As summarized in Table 2, the questionnaire covers four dimensions: accuracy, speed and convenience, error recognition and correction, and physical load or overall satisfaction. The tangram task excluded Wrist Rotation from the response options because Wrist Rotation was not applied to that task.

RCQ and TAAQ were treated as exploratory study-specific instruments. The distinct categorical TAAQ items were analyzed separately, rather than combined as a latent-scale score.

Finally, after completing all tasks, participants evaluated their overall experience with each interaction technique using the User Experience Questionnaire (UEQ) [34]. Unlike the task-specific questionnaires, the UEQ captures holistic perceptions of each interaction method, including pragmatic and hedonic qualities, and serves as a global assessment of user experience across the entire experiment.

### 3.5. Statistical Analysis

Statistical analyses used Python 3.14.3 [35], NumPy 2.4.3 [36], and SciPy 1.17.1 [37]. Workbook reading used openpyxl 3.1.5 [38]; analysis plots used Matplotlib 3.11.2 [39].

RCQ used each participant’s two-item mean. Single-factor aligned ranks [40] were analyzed by repeated-measures ANOVA with participant effects, GG correction, and rank-scale partial $\eta_{p}^{2}$. Significant task–dimension tests were followed by Holm-adjusted paired Wilcoxon tests (six pairs; three for tangram). The nine omnibus tests were exploratory without cross-dimension correction.

All 1200 trials contributed to participant mean log times. Task-wise repeated-measures ANOVAs used GG correction and partial $\eta_{p}^{2}$. Paired log-time *t*-tests (df=19) used separate within-task Holm families for VR pairs (six; three for tangram) and baseline contrasts (four; three for tangram). Exponentiated mean log differences give geometric time ratios; relative excess is 100(ratio−1). Absolute differences use paired arithmetic mean seconds.

Completion-time 95% CIs are marginal Student-*t* intervals; significance uses Holm-adjusted *p*-values, which may differ from unadjusted CI decisions. A paired Wilcoxon sensitivity analysis yielded the same significance decisions as the primary completion-time tests.

Internal consistency used RCQ Spearman–Brown coefficients [41] and UEQ standardized Cronbach’s alpha after coding to −3 to +3 [42]. TAAQ choices used exact multinomial tests (Holm across 20 items). Q2 speed choices were compared with measured fastest techniques using agreement, Wilson 95% CIs, Cohen’s κ [43], and fixed-marginal exact agreement tests (Holm across three tasks).

For n=20, 80% power sensitivity for two-sided paired tests was $|d_{z}|=0.660$ at α=0.05, and 0.784, 0.815, and 0.858 at fixed α=0.05/m for m=3, 4, 6 [44]. These strictest-Holm per-contrast thresholds are not prospective sample-size justification or family-wide power.

## 4. Usability Evaluation Results

A cross-design experiment involving three content-based VR tasks and four interaction techniques was conducted, yielding task-completion times and questionnaire responses for usability evaluation across conditions. The analyses in this section focus on comparing interaction techniques within each task type and examining their alignment with real-world task performance.

RCQ analyses used participant-level repeated measures as described in Section 3.5.

Subjective task-specific preferences were examined using the Technique-Appropriateness Assessment Questionnaire (TAAQ), which captured participants’ selections across dimensions of accuracy, speed and convenience, error handling, and physical load. Task completion time was analyzed to compare interaction efficiency across VR techniques and relative to real-world baselines.

Overall user experience was evaluated using the User Experience Questionnaire (UEQ) [34], covering six subscales: attractiveness, perspicuity, efficiency, dependability, stimulation, and novelty. Prior VR experience is reported descriptively; no experience-stratified UEQ inference is reported.

The following subsections present detailed results for each task. Task completion time and UEQ results are subsequently summarized and discussed in a dedicated subsection.

### 4.1. Discrete Task: Text Typing

**Figure 5:**
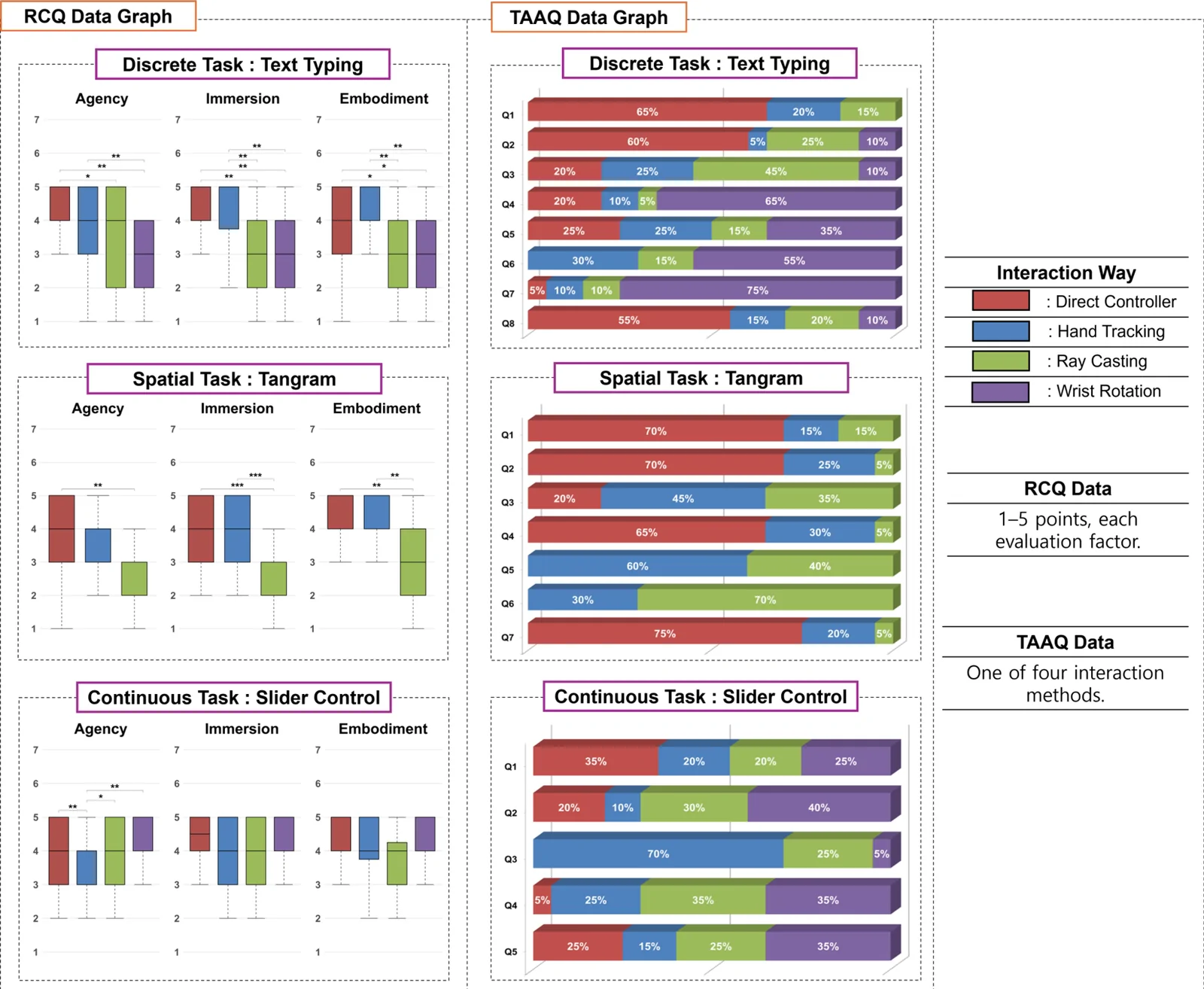
Overview of RCQ and TAAQ results. The left panels present RCQ scores for agency, immersion, and embodiment across the three tasks. The right panels show TAAQ response distributions for each question across the interaction techniques (three for tangram). RCQ boxes summarize pooled item responses (two items per dimension from 20 participants), whereas tests use the 20 participant-level two-item means. Brackets indicate Holm-adjusted paired Wilcoxon comparisons, shown only following a significant omnibus test (* *p* < 0.05, ** *p* < 0.01, *** *p* < 0.001).

This section reports results for the discrete text typing task, comparing the four interaction techniques using the three RCQ dimensions—agency, immersion, and embodiment—and subjective preferences from the Technique-Appropriateness Assessment Questionnaire (TAAQ). An overview of RCQ and TAAQ results is shown in Figure 5.

#### 4.1.1. RCQ Results

Technique effects were detected for agency (F(2.88,54.65)=10.93, $\eta_{p}^{2}=0.365$), immersion (F(2.63,50.00)=14.24, $\eta_{p}^{2}=0.428$), and embodiment (F(2.67,50.76)=16.18, $\eta_{p}^{2}=0.460$); all GG p<0.001.

- **Agency:** Direct Controller (M=4.33) scored higher than Raycasting (M=3.43; $V_{+}=132.5$, $p_{\mathrm{Holm}}=0.033$, $r_{B}=0.732$). Direct Controller and Hand Tracking (M=4.00) both exceeded Wrist Rotation (M=2.88; both $p_{\mathrm{Holm}}=0.004$). Remaining contrasts were non-significant.
- **Immersion:** Hand Tracking (M=4.28) and Direct Controller (M=4.10) scored higher than Raycasting (M=3.10; $p_{\mathrm{Holm}}\leq 0.007$). Wrist Rotation (M=2.90) was lower than both Hand Tracking and Direct Controller ($p_{\mathrm{Holm}}\leq 0.007$) but did not differ significantly from Raycasting.
- **Embodiment:** Hand Tracking (M=4.38) and Direct Controller (M=4.00) outperformed Raycasting (M=3.10; $p_{\mathrm{Holm}}\leq 0.022$). Wrist Rotation (M=3.15) scored lower than Hand Tracking and Direct Controller ($p_{\mathrm{Holm}}\leq 0.029$) and did not differ significantly from Raycasting.

#### 4.1.2. TAAQ Preferences

TAAQ responses indicated that Direct Controller was most frequently selected for accurate key input (Q1, 65%), fast typing speed (Q2, 60%), and overall satisfaction (Q8, 55%). Raycasting was most frequently selected for sentence visibility (Q3, 45%). Wrist Rotation was most frequently selected for error correction during typing (Q4, 65%; Q5, 35%), while also receiving the highest frequencies for manipulation errors (Q6, 55%) and postural fatigue (Q7, 75%). Hand Tracking did not receive the highest selection frequency for any individual item but showed relatively balanced distributions across multiple categories.

#### 4.1.3. Summary

In the text typing task, Direct Controller and Hand Tracking exhibited higher immersion and embodiment scores than Raycasting and Wrist Rotation; agency depended on the specific comparison. Direct Controller also received the highest selection frequencies for accuracy, speed, and overall satisfaction in the TAAQ. Raycasting showed relatively higher preference for sentence visibility, while Wrist Rotation was associated with both favorable error-correction preferences and increased reports of fatigue and control difficulty.

### 4.2. Spatial Task: Tangram

This section reports results for the spatial tangram task, comparing three interaction techniques using the RCQ dimensions (agency, immersion, and embodiment) and subjective preferences from the Technique-Appropriateness Assessment Questionnaire (TAAQ). An overview of the results is shown in Figure 5.

#### 4.2.1. RCQ Results

Technique effects were detected for agency (F(1.84,35.01)=7.05, $p_{\mathrm{GG}}=0.003$, $\eta_{p}^{2}=0.271$), immersion (F(1.64,31.18)=18.72, $\eta_{p}^{2}=0.496$), and embodiment (F(1.91,36.34)=15.33, $\eta_{p}^{2}=0.447$); the latter two GG p<0.001.

- **Agency:** Direct Controller (M=3.98) scored higher than Raycasting (M=2.90; $V_{+}=142.5$, $p_{\mathrm{Holm}}=0.005$, $r_{B}=0.863$). The DC–HT (M=3.30) and HT–RC contrasts were non-significant (both $p_{\mathrm{Holm}}=0.146$).
- **Immersion:** Direct Controller (M=3.85) and Hand Tracking (M=4.00) both yielded higher scores than Raycasting (M=2.43; $p_{\mathrm{Holm}}\leq 0.002$). No significant difference was found between Direct Controller and Hand Tracking, with Hand Tracking showing the highest mean.
- **Embodiment:** Direct Controller (M=4.23) and Hand Tracking (M=4.20) both outperformed Raycasting (M=3.08; $p_{\mathrm{Holm}}\leq 0.002$ for both), with no significant difference between the two.

#### 4.2.2. TAAQ Preferences

TAAQ responses indicated that Direct Controller was most frequently selected for precise placement (Q1, 70%), fast manipulation (Q2, 70%), ease of correcting misalignments (Q4, 65%), and overall satisfaction (Q7, 75%). Hand Tracking received the highest selection frequency for moving multiple pieces simultaneously (Q3, 45%) and was most frequently selected as prone to manipulation errors (Q5, 60%). These are subjective judgments rather than recorded error events. Raycasting was most frequently selected for postural fatigue (Q6, 70%) and received relatively fewer selections across accuracy- and efficiency-related items.

#### 4.2.3. Summary

In the tangram task, Direct Controller and Hand Tracking generally received higher RCQ scores than Raycasting across immersion and embodiment dimensions, with Direct Controller also showing higher agency scores. Preference data indicated that Direct Controller was most frequently selected for precision-, speed-, and satisfaction-related criteria, while Hand Tracking showed strengths in multi-object manipulation alongside more frequent perceived-error selections. Raycasting was more frequently associated with physical fatigue and lower preference across most criteria.

### 4.3. Continuous Task: Slider Control

This section reports results for the continuous slider control task, comparing four interaction techniques using the RCQ dimensions (agency, immersion, and embodiment) and subjective preferences from the Technique-Appropriateness Assessment Questionnaire (TAAQ). An overview of the results is shown in Figure 5.

#### 4.3.1. RCQ Results

Agency differed by technique (F(2.37,44.98)=8.56, $p_{\mathrm{GG}}<0.001$, $\eta_{p}^{2}=0.311$). Immersion (F(2.77,52.57)=2.37, $p_{\mathrm{GG}}=0.086$, $\eta_{p}^{2}=0.111$) and embodiment (F(2.36,44.75)=2.22, $p_{\mathrm{GG}}=0.112$, $\eta_{p}^{2}=0.105$) were non-significant, so no paired follow-up is reported for them.

- **Agency:** Direct Controller (M=4.03), Raycasting (M=4.05), and Wrist Rotation (M=4.28) all scored significantly higher than Hand Tracking (M=3.18; DC–HT: $V_{+}=105.0$, $p_{\mathrm{Holm}}=0.005$, $r_{B}=1.000$; RC–HT: $p_{\mathrm{Holm}}=0.029$; WR–HT: $p_{\mathrm{Holm}}=0.004$). No significant differences were observed among the three higher-scoring techniques.
- **Immersion:** No significant overall effect was found ($p_{\mathrm{GG}}=0.086$); means were Direct Controller (M=4.30), Hand Tracking (M=4.00), Raycasting (M=3.73), and Wrist Rotation (M=3.98), without follow-up pairwise claims.
- **Embodiment:** Direct Controller (M=4.38), Hand Tracking (M=4.08), Raycasting (M=3.83), and Wrist Rotation (M=4.23) differed descriptively, but the overall effect was non-significant ($p_{\mathrm{GG}}=0.112$); no follow-up pairwise inference is reported.

#### 4.3.2. TAAQ Preferences

TAAQ responses indicated that Direct Controller was most frequently selected for precise slider control (Q1, 35%) and tied with Raycasting for the second-highest satisfaction selection frequency (Q5, 25% each; Wrist Rotation, 35%). Wrist Rotation was most frequently selected for fast manipulation (Q2, 40%). Hand Tracking was most frequently selected as prone to manipulation errors (Q3, 70%), indicating perceived error susceptibility rather than a measured error rate. Raycasting tied with Wrist Rotation for postural fatigue (Q4, 35% each) and ranked second for perceived speed (Q2, 30%).

#### 4.3.3. Summary

In the slider control task, Direct Controller, Raycasting, and Wrist Rotation received higher agency scores than Hand Tracking; no overall immersion or embodiment effect was detected. Preference data showed that Direct Controller was more frequently selected for precision-related criteria, while Wrist Rotation was more frequently selected for speed-related criteria, alongside higher reports of physical fatigue. Hand Tracking was more frequently associated with manipulation errors, and Raycasting showed intermediate patterns across most preference dimensions.

### 4.4. Task Completion Time Comparison by Interaction Technique

**Figure 6:**
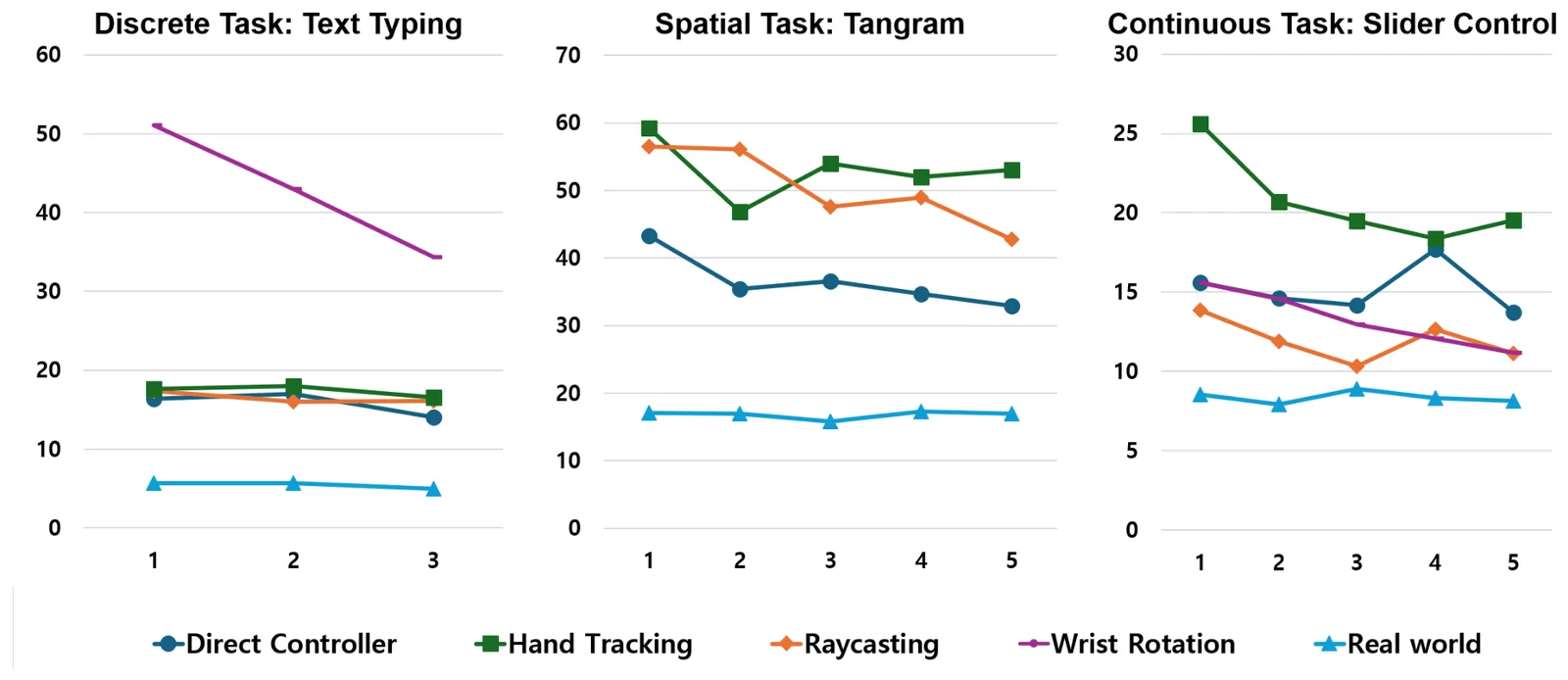
Task completion time comparison graph. The graph illustrates the changes in completion time across three typing trials and five tangram/slider trials. Controller Direct, Hand Direct, and Real Environment in the legend denote DC, HT, and RW, respectively.

Task completion times were compared across interaction techniques for each task type. The comparison included real-world interaction times—obtained from the same participants performing the tasks while the experimenter measured their times using a timer—as well as completion times for the four VR interaction methods. Figure 6 and Table 3 summarize the results.

Technique effects were detected in each task on participant mean log times: text typing (F(2.18,41.50)=147.82, $p_{\mathrm{GG}}<0.001$, $\eta_{p}^{2}=0.886$); tangram (F(2.00,37.91)=38.90, $p_{\mathrm{GG}}<0.001$, $\eta_{p}^{2}=0.672$); slider control (F(2.79,52.92)=40.36, $p_{\mathrm{GG}}<0.001$, $\eta_{p}^{2}=0.680$).

**Discrete Task (Text Typing).** The average task completion time in the real world was 5.47 s. In VR, Direct Controller (15.78 s), Raycasting (16.52 s), and Hand Tracking (17.40 s) did not differ significantly after Holm correction (DC–HT: p=0.093; DC–RC: 0.581; HT–RC: 0.169). Wrist Rotation resulted in longer durations (42.80 s on average; all corrected contrasts p<0.001), although a clear reduction in completion time was observed across repeated trials, decreasing from approximately 50 s to the mid-30-s range.

**Spatial Task (Tangram).** The average completion time in the real world was 16.81 s. In VR, Direct Controller (36.57 s) was faster than Raycasting (50.37 s) and Hand Tracking (53.01 s; both $p_{\mathrm{Holm}}<0.001$; DC/RC time ratio 0.737 [95% CI 0.672–0.808], DC/HT 0.706 [0.646–0.772]). The HT–RC contrast was non-significant ($p_{\mathrm{Holm}}=0.333$). Both Direct Controller and Raycasting showed decreasing completion times over trials, and Hand Tracking remained relatively slower across repetitions.

**Continuous Task (Slider Control).** The average real-world completion time was 8.36 s. In VR, Raycasting (11.97 s) was faster than Direct Controller (15.15 s; DC/RC ratio 1.218 [95% CI 1.108–1.339], $p_{\mathrm{Holm}}=0.001$). Wrist Rotation averaged 13.27 s; DC–WR and RC–WR contrasts were non-significant ($p_{\mathrm{Holm}}=0.075$ and 0.098). Hand Tracking (20.72 s) was slower than every alternative (all $p_{\mathrm{Holm}}<0.001$). Wrist Rotation exhibited a reduction in completion time across repeated trials, approaching Raycasting performance in later trials.

Completion times varied across trials, but stimulus variation, including increasing typing length, prevents interpreting these trends as isolated learning effects. These within-VR observations do not establish whether the order of the real-world and VR phases affected performance. Because participant-level sequences were not recorded and the numerical tangram tolerance could not be verified, the reported technique differences and real-world gaps may include uncontrolled order effects, and tangram times reflect the implemented acceptance criterion rather than a defined placement accuracy.

The smallest estimated gaps were DC for typing (+10.31 s; +191.0%), DC for tangram (+19.76 s; +114.6%), and RC for slider control (+3.61 s; +38.1%). These are point-estimate rankings: typing DC was not distinguishable from HT or RC, and slider RC was not distinguishable from WR after correction. For each task, all evaluated VR techniques remained slower than the real-world baseline (within-task Holm p<0.001).

### 4.5. Precision and Reliability of UEQ-Based User Experience Ratings

We quantified the precision of UEQ scale means using 95% confidence intervals (CIs) for each technique and subscale. CI half-widths (margins of error) use $1.96\;SD/\sqrt{20}$; they describe uncertainty in estimated means rather than internal consistency. Table 4 summarizes these results.

Intervals included zero for DC Novelty and WR Perspicuity and Efficiency. Standardized Cronbach’s alpha for each technique and subscale is reported in Table 5. Coefficients below 0.70 (UEQ: WR and DC Dependability, RC Perspicuity, and HT Efficiency; RCQ: DC and HT immersion in the spatial task, HT immersion and embodiment and DC embodiment in the continuous task) indicate low internal consistency, and results on these scales are interpreted with caution.

### 4.6. Dimension-Wise Comparison of User Experience Scores

**Figure 7:**
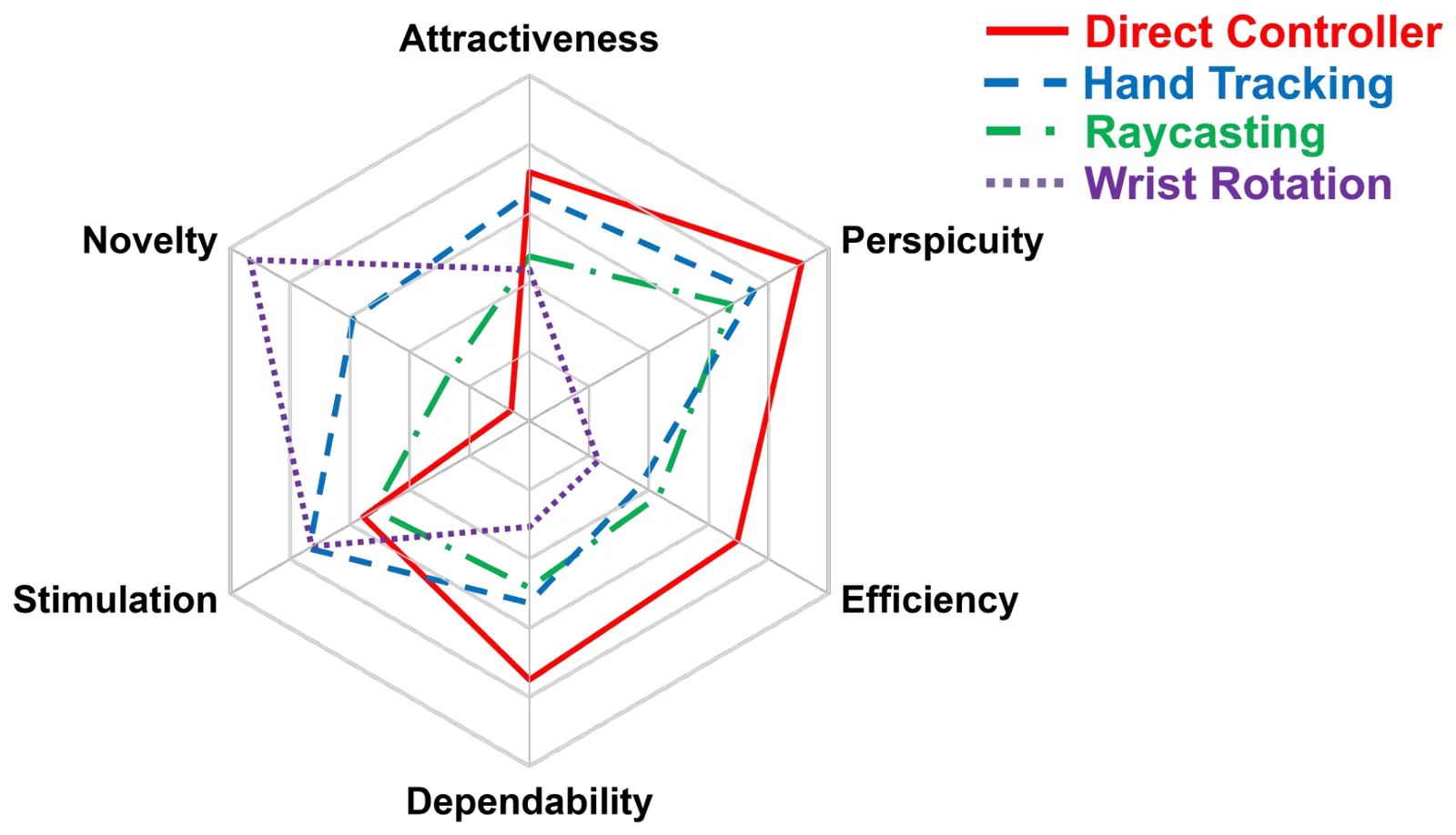
Radar charts of the UEQ scales for each interaction technique, visualized across six dimensions: Attractiveness, Perspicuity, Efficiency, Dependability, Stimulation, and Novelty.

Descriptive UEQ dimension scores for each interaction technique were visualized using a radar chart to compare perceived experiential characteristics across the six UEQ dimensions (Figure 7).

The four interaction techniques exhibited distinct profiles across the six UEQ dimensions:

**Direct Controller.** Direct Controller showed consistently high ratings in pragmatic quality dimensions, particularly Perspicuity, Efficiency, and Dependability, indicating a clear, reliable, and controllable interaction experience. In contrast, its lower scores in Stimulation and Novelty suggest that users perceived it as familiar and functionally stable rather than exciting or innovative.

**Hand Tracking.** Hand Tracking achieved the highest observed Stimulation mean (1.838) and the second-highest Novelty mean (1.475). However, comparatively lower ratings in Efficiency and Dependability indicate variability in task performance and perceived stability.

**Wrist Rotation.** Wrist Rotation had the highest observed Novelty mean (2.325) and a Stimulation mean of 1.813. Nevertheless, lower scores in Perspicuity, Efficiency, and Dependability suggest challenges related to intuitiveness and operational reliability.

**Raycasting.** Raycasting demonstrated moderate performance across most UEQ dimensions, with relatively higher ratings in Perspicuity, indicating ease of understanding. However, lower Novelty scores suggest that it was perceived as a conventional and less innovative interaction technique.

**Overall.** Overall, the UEQ results reveal a clear distinction between pragmatic and hedonic user experience characteristics across interaction techniques. Direct Controller consistently excelled in pragmatic quality dimensions, whereas Hand Tracking and Wrist Rotation showed strengths in hedonic dimensions such as Stimulation and Novelty. These descriptive profiles suggest trade-offs between perceived usability and engagement; no paired UEQ significance tests were conducted.

### 4.7. Measurement Checks for the RCQ and TAAQ

RCQ two-item Spearman–Brown coefficients for each task, technique, and dimension are also reported in Table 5. Twelve of 20 TAAQ items showed non-uniform choices after Holm correction. These preference-distribution tests do not establish validity. Q2 speed choices matched the fastest measured technique for 30% of participants in typing (95% CI 14.5–51.9%), 65% in tangram (43.3–81.9%), and 45% in slider control (25.8–65.8%). Chance-adjusted agreement was κ=−0.065,−0.085,0.185 for typing, tangram, and slider control, respectively (fixed-marginal exact Holm p=1.000,1.000,0.907).

## 5. Discussion

Building upon the task taxonomy established in our previous systematic mapping study [11], this study investigated how four representative VR interaction techniques—Direct Controller, Hand Tracking, Raycasting, and Wrist Rotation—perform across discrete, spatial, and continuous task types, grounded by within-participant comparison with corresponding real-world tasks. The results reveal clear and systematic differences not only in task performance but also in experiential qualities, showing that technique suitability varied across the three tested tasks.

Direct Controller was faster than HT and RC for tangram, while typing differences among DC, HT, and RC were non-significant; it also received favorable ratings in subjective user experience dimensions related to pragmatic quality, including efficiency, dependability, and perspicuity. This stability may be attributed to its mature hardware design, physical affordances, and well-established control mappings, which closely resemble real-world tool use. These characteristics may allow users to transfer prior motor skills with minimal adaptation, supporting predictable and reliable interaction across the tested tasks.

Hand Tracking, in contrast, exhibited a distinct performance profile. Its completion times were longer than DC for tangram and every alternative for sliders. Hand Tracking had the highest descriptive Stimulation mean, whereas WR had the highest Novelty mean. This suggests that hand tracking excels in delivering expressive and embodied interaction experiences, even though current sensing limitations, occlusion issues, and the absence of physical constraints may hinder fine-grained control. These findings, considered alongside prior work on naturalness and precision in controller-free interaction [3,14], suggest potential value for experiential, exploratory, or engagement-oriented VR applications, with the measured time costs taken into account.

Raycasting occupied an intermediate position across most task conditions. Its strengths lie in remote selection and clear visual feedback, which supported reasonable efficiency in discrete selection and continuous adjustment tasks.

For slider control, Raycasting had the shortest observed mean completion time, although the RC–WR contrast was non-significant after correction. However, lower ratings in engagement-related dimensions indicate that raycasting may feel indirect or detached, especially during sustained manipulation or precision-sensitive interaction.

This suggests that raycasting may be suited for navigation, menu interaction, and distant object selection; the slider results also support considering it for time-prioritized continuous adjustment.

A key contribution of this work is the inclusion and systematic evaluation of Wrist Rotation as a task-specific interaction technique. Although wrist rotation has been explored in prior studies primarily for menu navigation or auxiliary input, its role has remained underdefined in comparative VR interaction research. For the tested slider task, WR warrants consideration alongside its time cost and perceived fatigue. Its time contrasts against RC and DC were non-significant after correction, which does not establish equivalence; RC and WR each received 35% of slider-fatigue selections. At the same time, its limitations—most notably the lack of positional input in the implemented mapping and reported ergonomic concerns—limit its use for the spatial manipulation task tested here. These findings clarify that wrist rotation should be understood not as a general-purpose interaction method, but as a complementary, task-specific technique whose time cost and perceived fatigue should be considered for the intended control scenario.

Real-world task performance provides a participant-specific reference for completion-time gaps under the evaluated setups. The observed disparities, particularly in spatial manipulation, describe differences between the physical tasks and the tested VR implementations, rather than an isolated cost of VR.

WR typing took 27.03 s longer per sentence than DC (95% CI 19.27–34.79), HT tangram took 16.44 s longer per puzzle than DC (10.01–22.87), and DC slider control took 3.19 s longer than RC (1.20–5.17). Costs accumulate with repetition; no application-specific minimum important difference was prespecified.

**Limitations and Multimodal Interaction Considerations.**

A limitation of this study is the relatively small sample size of 20 participants. All were aged 20–25, and 18 had no or limited VR experience. Although the study identified task-dependent differences across the evaluated interaction techniques, the limited sample size may reduce statistical power for detecting subtle effects and constrain the generalizability of the findings. Therefore, the results should be interpreted with caution, particularly when extending the observed patterns to broader user populations. Future studies with larger and more diverse participant samples are needed to further validate these findings.

Each task category was represented by one task implementation, so the observed technique profiles cannot be generalized to all discrete, spatial, or continuous tasks.

Balancing real-world-first and VR-first sequences does not by itself establish the absence of learning or carryover effects. Participant-level environment-order assignments and VR technique sequences were unavailable in the reanalysis files, so sequence effects could not be tested or adjusted for. Different stimuli, input devices, and manual real-world versus automatic VR timing also limit baseline comparisons; observer reaction delay and stimulus equivalence were not quantified.

Objective error rates and correction counts could not be reconstructed, and the numerical tangram tolerance remains unverified. TAAQ choices cannot establish objective accuracy differences.

Internal consistency does not establish the RCQ factor structure or validate all TAAQ items. The speed-item check showed no significant departure from fixed-marginal chance agreement. Questionnaire findings are therefore interpreted as exploratory, item-based descriptions of perceived experience and preferences; broader construct interpretations remain unverified. Further validation should examine item comprehension, the proposed RCQ dimensions, and relationships with theoretically aligned measures in an independent sample.

Another limitation is that the interaction techniques were evaluated as isolated modalities, whereas recent VR head-mounted displays, as part of the broader XR device evolution, increasingly support multimodal natural interaction, including eye tracking, hand input, and voice commands. Such capabilities allow users to flexibly combine or switch between input modalities depending on task demands.

However, the intentional focus on unimodal interaction in this study was necessary to isolate the fundamental relationships between task characteristics and interaction techniques. Evaluating each technique independently enabled a clearer attribution of performance and experiential effects, which would be difficult to disentangle in fully multimodal settings. From this perspective, the present findings can be interpreted as foundational building blocks for future multimodal interaction design in VR.

Future work will extend this framework by systematically investigating multimodal combinations—such as gaze-assisted selection or adaptive modality switching—while preserving task awareness and ergonomic constraints.

## 6. Conclusions and Future Work

Extending our previous systematic mapping study [11], which identified representative VR task categories and interaction techniques, this study experimentally compared four representative VR interaction techniques—Direct Controller, Hand Tracking, Raycasting, and Wrist Rotation—across three representative task types: discrete (text typing), spatial (tangram manipulation), and continuous (slider control). By incorporating corresponding real-world tasks as a participant-specific performance baseline, the study provides a grounded evaluation framework that clarifies technique–task suitability beyond VR-only comparisons.

Direct Controller was faster than Hand Tracking and Raycasting for tangram and had the highest observed UEQ pragmatic-quality means. Typing times among these three techniques did not differ significantly after correction, whereas Wrist Rotation was slower. Raycasting had the smallest estimated slider gap from real-world performance; its time difference from Wrist Rotation was non-significant. Hand Tracking received higher immersion and embodiment ratings than Raycasting in typing and tangram, alongside longer times for tangram and slider control. Collectively, these results reinforce the view that VR interaction effectiveness is inherently task-dependent.

Task-specific accuracy and error measures are needed alongside completion time to assess technique–task suitability more fully. Extending the experimental software to log erroneous inputs, corrective actions, and placement deviations would support this broader evaluation.

Future work will extend this research in several directions. First, a broader range of tasks within each category will be explored to further validate the generalizability of the observed patterns. Second, multimodal interaction strategies within VR—such as gaze-assisted selection, voice-supported commands, and haptic augmentation—will be investigated to address the limitations of single-modality approaches. Third, individual differences—including prior VR experience, age, and motor skill variability—will be analyzed to inform adaptive and personalized interaction design. Finally, long-term studies will examine sustained use factors such as physical fatigue, learning effects, and evolving user expectations, and the proposed evaluation framework may be adapted to augmented and mixed reality environments to support broader comparative interaction research.

## Figures and Tables

**Table 1:** Reality-Comparison Questionnaire (RCQ): 6 items rated on a 5-point Likert scale (1 = Strongly Disagree, 5 = Strongly Agree).

Category	Question Item
Agency	• I felt that my intentions (e.g., touch, grab) were accurately reflected in the virtual environment.
	• I felt that I was fully in control of the virtual interface’s actions.
Immersion	• I felt as if I was interacting with virtual objects directly, without a mediating device.
	• Interacting with virtual objects using the given method felt natural.
Embodiment	• The virtual interface (e.g., Direct Controller, Hand Tracking) felt like my real hand.
	• It felt as if my real hand was located where the virtual interface appeared.

**Table 2:** Technique-Appropriateness Assessment Questionnaire (TAAQ). For each item, participants selected the single interaction method (Direct Controller, Hand Tracking, Raycasting, Wrist Rotation *) that best matched the item (* Wrist Rotation was not applied to the tangram task).

Task	Category	Question Item
Text Typing	Accuracy	Q1. Which method allowed for the most accurate key input?
	Speed Convenience	Q2. Which method was most suitable for achieving fast typing speed?
		Q3. Which method made it easier to check the target sentence while typing?
	Error Handling	Q4. Which method made it easiest to correct typos immediately during typing?
		Q5. Which method made it easiest to correct errors identified after completion?
		Q6. Which method was most prone to operational errors?
	Physical Load Satisfaction	Q7. Which method caused the most postural fatigue?
		Q8. Which method was most satisfying for typing overall?
Tangram	Accuracy	Q1. Which method was best for precise movement, rotation, and placement of pieces?
	Speed Convenience	Q2. Which method enabled fast movement and alignment of pieces?
		Q3. Which method was efficient for moving multiple pieces simultaneously?
	Error Handling	Q4. Which method made it easy to correct misaligned or misoriented pieces?
		Q5. Which method felt prone to manipulation errors?
	Physical Load Satisfaction	Q6. Which method caused the most postural fatigue?
		Q7. Which method was most satisfying overall for the task?
Slider Control	Accuracy	Q1. Which method allowed for precise control of playback, volume, and brightness?
	Speed	Q2. Which method enabled the fastest adjustment of playback, volume, and brightness?
	Error Recognition	Q3. Which method was most prone to manipulation errors?
	Physical Load Satisfaction	Q4. Which method caused the most postural fatigue?
		Q5. Which method was most satisfying overall for operating the video player?

**Table 3:** Task completion times (seconds) across interaction techniques and task types.

Task Type	Trial/Measure	DC ^1^	HT	RC	WR	RW
**Discrete**	1	16.35	17.62	17.38	51.09	5.72
	2	16.97	18.01	16.05	43.00	5.72
	3	14.01	16.57	16.14	34.33	4.97
	**Avg.**	**15.78**	**17.40**	**16.52**	**42.80**	**5.47**
	Difference (s)	10.31 [8.50, 12.13]	11.94 [10.18, 13.69]	11.06 [8.45, 13.67]	37.34 [29.80, 44.88]	—
	Increase (%)	191.0 [154.0, 233.4]	224.8 [183.5, 272.1]	201.0 [160.2, 248.2]	672.7 [569.7, 791.4]	—
**Spatial**	1	43.30	59.19	56.46	N/A	17.10
	2	35.40	46.85	56.05	N/A	16.96
	3	36.56	54.01	47.55	N/A	15.82
	4	34.68	51.98	48.96	N/A	17.26
	5	32.93	53.02	42.82	N/A	16.93
	**Avg.**	**36.57**	**53.01**	**50.37**	N/A	**16.81**
	Difference (s)	19.76 [16.10, 23.42]	36.20 [28.65, 43.75]	33.55 [26.80, 40.31]	N/A	—
	Increase (%)	114.6 [96.5, 134.4]	203.8 [174.3, 236.4]	191.3 [160.7, 225.5]	N/A	—
**Continuous**	1	15.59	25.61	13.85	15.59	8.53
	2	14.60	20.67	11.89	14.55	7.92
	3	14.15	19.49	10.33	12.97	8.90
	4	17.69	18.34	12.64	12.06	8.29
	5	13.73	19.51	11.13	11.17	8.14
	**Avg.**	**15.15**	**20.72**	**11.97**	**13.27**	**8.36**
	Difference (s)	6.80 [4.21, 9.39]	12.37 [9.78, 14.95]	3.61 [2.62, 4.60]	4.91 [3.00, 6.82]	—
	Increase (%)	68.1 [47.1, 92.3]	128.1 [106.3, 152.2]	38.1 [27.2, 49.8]	49.0 [35.0, 64.5]	—

Brackets show marginal 95% confidence intervals from 20 complete participant pairs per contrast. Difference (s) is the paired mean VR minus RW time. Increase (%) is the percentage increase over RW, estimated from paired geometric time ratios. ^1^ DC = Direct Controller, HT = Hand Tracking, RC = Raycasting, WR = Wrist Rotation, RW = Real World.

**Table 4:** Mean scores and 95% confidence intervals (CI) for each interaction technique in the UEQ subscales.

Interaction Technique	Subscales	Mean	Std. Dev.	CI Half-Width	CILower	CIUpper
Direct Controller	Attractiveness Perspicuity	1.800 2.275	0.873 0.831	0.383 0.364	1.417 1.911	2.183 2.639
	Efficiency Dependability	1.738 1.875	0.975 0.772	0.427 0.338	1.310 1.537	2.165 2.213
	Stimulation Novelty	1.388 0.150	1.193 1.633	0.523 0.716	0.864 −0.566	1.911 0.866
Wrist Rotation	Attractiveness Perspicuity	1.092 0.375	1.254 1.324	0.549 0.580	0.542 −0.205	1.641 0.955
	Efficiency Dependability	0.575 0.763	1.509 1.093	0.661 0.479	−0.086 0.284	1.236 1.241
	Stimulation Novelty	1.813 2.325	1.076 0.744	0.471 0.326	1.341 1.999	2.284 2.651
Raycasting	Attractiveness Perspicuity	1.192 1.688	1.379 0.914	0.604 0.400	0.587 1.287	1.796 2.088
	Efficiency Dependability	1.088 1.213	1.401 1.234	0.614 0.541	0.474 0.672	1.701 1.753
	Stimulation Novelty	1.300 0.675	1.202 1.419	0.527 0.622	0.773 0.053	1.827 1.297
Hand Tracking	Attractiveness Perspicuity	1.650 1.875	1.273 0.998	0.558 0.438	1.092 1.437	2.208 2.313
	Efficiency Dependability	0.925 1.313	1.230 1.328	0.539 0.582	0.386 0.731	1.464 1.894
	Stimulation Novelty	1.838 1.475	1.104 1.455	0.484 0.638	1.354 0.837	2.321 2.113

**Table 5:** Internal consistency by condition. UEQ: standardized Cronbach’s α for each technique and subscale. RCQ: two-item Spearman–Brown coefficient for each task, technique, and dimension. ^†^ Below 0.70.

UEQ		Attr.		Persp.	Eff.	Dep.		Stim.		Nov.	
DC		0.895		0.811	0.820	0.616 ^†^		0.862		0.914	
HT		0.883		0.724	0.691 ^†^	0.715		0.771		0.851	
RC		0.887		0.675 ^†^	0.801	0.748		0.843		0.868	
WR		0.863		0.757	0.886	0.440 ^†^		0.888		0.900	
**RCQ (Task Type)**			**Technique**		**Agency**	**Immersion**			**Embodiment**		
**Discrete**			DC		0.859	0.790			0.795		
			HT		0.779	0.802			0.948		
			RC		0.898	0.712			0.863		
			WR		0.792	0.806			0.789		
**Spatial**			DC		0.926	0.694 ^†^			0.754		
			HT		0.766	0.627 ^†^			0.754		
			RC		0.863	0.823			0.913		
**Continuous**			DC		0.746	0.863			0.571 ^†^		
			HT		0.829	0.500 ^†^			0.629 ^†^		
			RC		0.895	0.770			0.780		
			WR		0.813	0.713			0.771		

## Data Availability

The datasets and analysis code supporting the findings of this study are publicly available in the Zenodo repository at https://doi.org/10.5281/zenodo.21357775.
